# Choosing the Right Treatment Option for the Right R/M HNSCC Patient: Should We Adhere to PFE for First-Line Therapy?

**DOI:** 10.3389/fonc.2021.715297

**Published:** 2021-07-20

**Authors:** Katharina Lübbers, Mykola Pavlychenko, Theresa Wald, Susanne Wiegand, Andreas Dietz, Veit Zebralla, Gunnar Wichmann

**Affiliations:** Department of Otorhinolaryngology, Head and Neck Surgery, University Hospital Leipzig, Leipzig, Germany

**Keywords:** head and neck cancer, head and neck squamous cell carcinoma, palliative chemotherapy, first-line therapy, recurrent/metastatic head and neck squamous cell carcinoma, multivariate Cox proportional hazard regression, outcome research, p16+ oropharyngeal cancer

## Abstract

**Background:**

The landmark EXTREME trial established cisplatin, 5-fluorouracil and cetuximab (PFE) as first-line chemotherapy (1L-ChT) for recurrent/metastatic head and neck squamous cell carcinoma (R/M HNSCC). We were interested in outcome differences of R/M HNSCC in 1L-ChT and factors influencing outcome in certain subgroups, especially patients receiving PFE, and the value of PFE compared to other 1L-ChT regimens to provide real world evidence (RWE).

**Methods:**

For this retrospective monocentric study, 124 R/M HNSCC patients without curative surgical or radiotherapy options receiving at least one cycle of 1L-ChT were eligible. We analyzed their outcome using Kaplan-Meier plot and Cox regression to identify predictors for prolonged survival.

**Results:**

Subgroups benefiting significantly from PFE were patients suffering from an index HNSCC outside the oropharynx. The PFE regimen proved to be superior to all other 1L-ChT regimens in clinical routine. Significant outcome differences between PFE treatment within or outside controlled trials were not seen.

**Conclusion:**

This retrospective analysis provides RWE for factors linked to improved outcome. Subgroup analyses highlight the lasting value of PFE among the growing spectrum of 1L-ChT. Importantly, fit smokers with high level alcohol consumption benefit from PFE; considering the patient’s lifestyle factors, PFE should not be ignored in decision-making.

## Introduction

Squamous cell carcinoma of the head and neck (HNSCC) is an entity with growing importance, in clinical but also in research settings. According to the EUROCARE-5 trial (1), there were 238,608 cases recorded from 1999 to 2007 in Europe. Five-years overall survival (OS) for all HNSCC entities was 42.2% (95% confidence interval, 95%CI: 41.5–42.9%), ranging from 25.5% for oropharynx to 61.1% for larynx cancer. At initial diagnosis of HNSCC, 54.0% of all HNSCC were classified as UICC IV due to regional or distant metastasis. According to the NCCN Guidelines for Head and Neck Cancer (2018) (2), curative therapy is considered appropriate until UICC IVB, whereas detection of distant metastasis (M1 defining stage IVC) means loss of curative treatment options advising switch to systemic treatment and palliative care (with the only exception of resectable solitary M1). The same applies to recurrent locally advanced HNSCC after radiotherapy without resectability. While there are certain therapy algorithms for HNSCC in curable stages, only a few approved options for first-line chemotherapy and other systemic first-line therapies (altogether summarized under the abbreviation 1L-ChT) are available in case of R/M HNSCC following the NCCN guidelines from 2018. Since publication of the landmark EXTREME trial (3), treatment with up to six cycles of cisplatin, 5-fluorouracil and cetuximab (PFE), became standard 1L-ChT in R/M HNSCC. After the KEYNOTE-048 trial (4), this standard was recommended being replaced by a stratified 1L-ChT according to programmed death ligand 1 (PD-L1) expression assessed by combined positivity score (CPS). According to immune histopathology, PFE remains a standard of care for patients with a CPS <1, whereas patients with a CPS ≥20 should be treated with pembrolizumab mono and patients with CPS ≥1 and <20 should receive cisplatin/5-fluoruracil/pembrolizumab (5).

Prior trials often used PFE as control arm (6–9), but new 1L-ChT options superior to PFE have not yet been identified or been established based on lower toxicity. In the course of precision medicine and decision-making for stratified therapy regimens leading to a more individualized or even personalized treatment, new therapy options became eligible for specific groups of patients as second-line therapy (2L-ChT) or 1L-ChT for patients not eligible for PFE (frail patients and/or insufficient kidney or liver function). We were interested in the outcome of PFE *versus* the other 1L-ChT and predictors for good outcome after PFE therapy and consequentially aimed on defining groups of patients that still benefit the most from PFE as part of a widened spectrum of therapy options.

## Materials and Methods

### Study Population and Patient Samples

**Figure 1:**
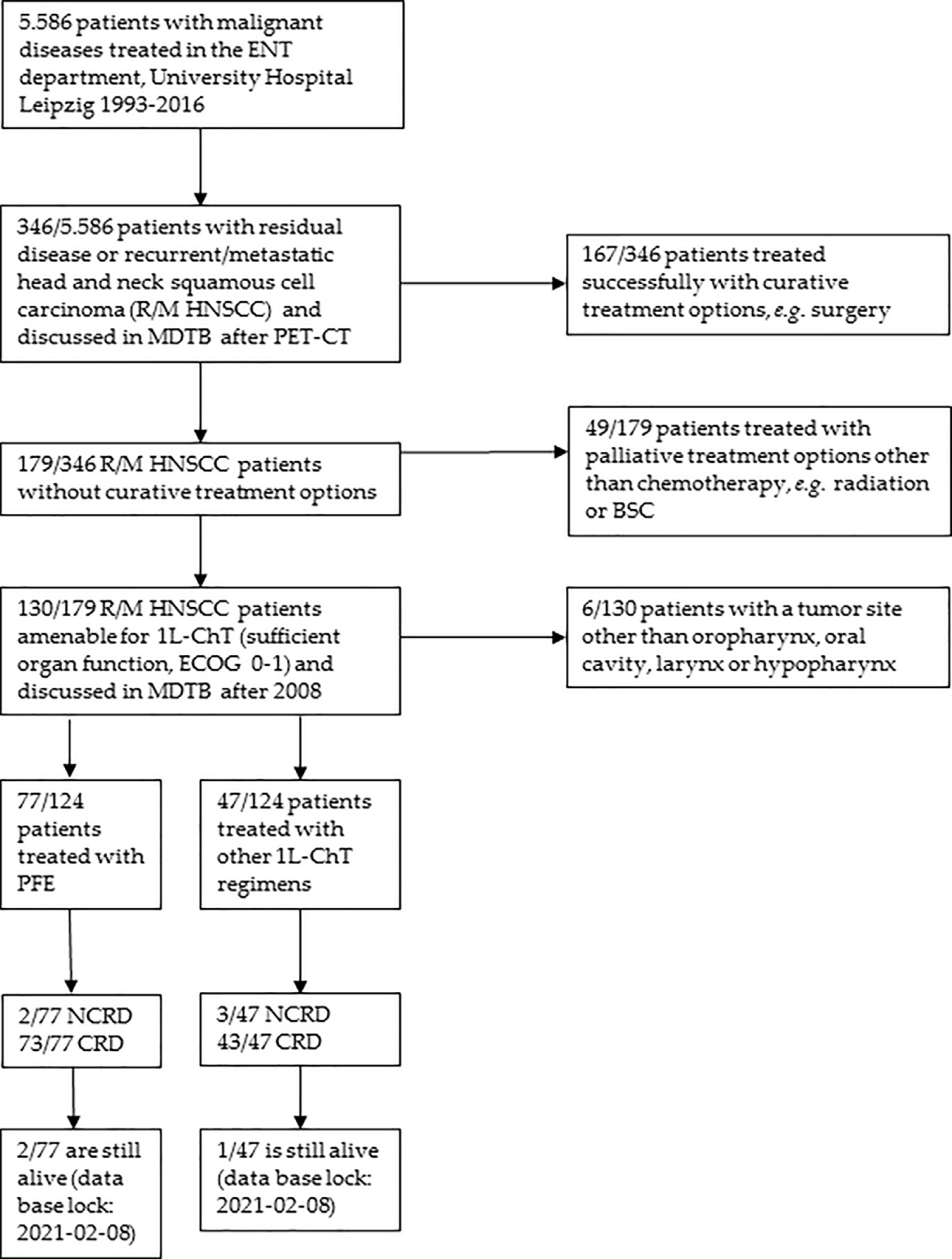
CONSORT diagram showing the selection criteria of recurrent/metastatic head and neck squamous cell carcinoma (R/M HNSCC) patients of the two cohorts compared. ECOG, Eastern Cooperative Oncology Group performance score; BSC, Best Supportive Care; MDTB, multi-disciplinary tumor board; NCRD, non cancer-related death; CRD, cancer-related death.

Eligible for the study were patients with pathological confirmed R/M HNSCC treated in the University Hospital Leipzig with data recorded in the Microsoft Access^®^ tumor database (TDB) of the ENT department, comprising data of all patients diagnosed with a malignant disease since 1990, and data taken from the hospital’s electronic health records. **Figure 1** shows the selection of patients for analyses according to the CONSORT recommendations. Among 346 R/M HNSCC patients presented to the multidisciplinary tumor board (MDTB; see below), 130 R/M HNSCC without curative treatment option were subjected to systemic therapy and received at least one cycle 1L-ChT. To prevent any inconsistency based on minor R/M HNSCC subgroups, patients with primary HNSCC localized in the nasopharynx (ICD10-C11), or nasal cavity (ICD-10-C30 and C31) were excluded from the present analyses resulting in a sample of 124 patients (**Table 1**). Pathological reports were available for all 124 patients. All resected specimen underwent pathological examination, and hematoxylin–eosin (HE) staining revealed squamous cell carcinoma histology. A sub-cohort of patients participated in a study approved by our Ethics Committee (votes 201-10-12072010 and 202-10-12072010).

**Table 1:** Clinical and epidemiological characteristics, diagnostic procedures and treatment as well as various survival measures for 5-years outcome of recurrent/metastatic head and neck squamous cell carcinoma (R/M HNSCC) patients of the two subgroups, PFE and other 1L-ChT.

Characteristics		Number of Patients (%)						
		Total	*N* = 124	PFE	*N* = 77	other 1L-ChT	*N* = 47	*P*-value*
Age at inital	<50	29	(23.4)	16	(20.8)	13	(27.7)	0.272
diagnosis, years	50–60	48	(38.7)	35	(45.5)	13	(27.7)	
	60–70	40	(32.3)	22	(28.6)	18	(38.3)	
	>70	7	(5.6)	4	(5.2)	3	(6.4)	
	median (IQR)	56.7	(50.2-63.7)	56.6	(50.2–63.1)	58.4	(49.4–64.1)	0.592
Age at 1L-ChT,	<50	19	(15.3)	11	(14.3)	8	(17.0)	0.199
years	50–59	50	(40.3)	36	(46.8)	14	(29.8)	
	60–69	45	(36.3)	26	(33.8)	19	(40.4)	
	>70	10	(8.1)	4	(5.2)	6	(12.8)	
	median (IQR)	58.8	(53.2–65.6)	58.1	(53.1–65.5)	61.0	(54.1–66.4)	0.279
ECOG	0–1	123	(99.2)	77	(100.0)	46	(97.9)	0.199
	2	1	(0.8)	0		1	(2.1)	
Sex	Male	105	(84.7)	67	(63.8)	38	(80.9)	0.355
	Female	19	(15.3)	10	(13.0)	9	(19.1)	
Alcohol, status	Missing	7	(5.6)	5	(6.5)	2	(4.3)	0.991
	Never	15	(12.1)	9	(11.7)	6	(12.8)	
	Former	13	(10.5)	8	(10.4)	5	(10.6)	
	Current	89	(71.8)	55	(71.4)	34	(72.3)	
Alcohol, (g/d)	Missing	7	(5.6)	5	(6.5)	2	(4.3)	0.999
	0 g/d	15	(12.8)	9	(11.7)	6	(12.8)	
	<30 g/d	36	(30.8)	22	(28.6)	14	(29.8)	
	30–60 g/d	29	(24.8)	18	(23.4)	11	(23.4)	
	>60 g/d	37	(31.6)	23	(29.9)	14	(29.8)	
Tobacco smo-	Missing	5	(4.0)	4	(5.2)	1	(2.1)	0.490
king, status	Never	13	(10.5)	6	(7.8)	7	(14.9)	
	Former	24	(19.4)	15	(19.5)	9	(19.1)	
	Current	82	(66.1)	52	(67.5)	30	(63.8)	
Tobacco	Missing	7	(5.6)	4	(5.2)	3	(6.4)	0.489
smoking history,	<30 py	59	(47.6)	35	(45.5)	24	(51.1)	
pack years	>30 py	58	(46.8)	38	(49.4)	20	(42.6)	
Localization	L-/HPSCC	31	(25.0)	17	(22.1)	14	(29.8)	0.053
	OSCC	45	(36.3)	23	(29.9)	22	(46.8)	
	OPSCC	42	(33.9)	32	(41.6)	10	(21.3)	
	other	6	(4.6)	5	(6.5)	1	(2.1)	
p16 status	p16 positive	17	(13.7)	13	(16.9)	4	(8.5)	0.188
	p16 negative	107	(86.3)	64	(83.1)	43	(91.5)	
HPV status	HPV positive	15	(12.1)	12	(15.6)	3	(6.4)	0.127
	HPV negative	109	(87.9)	65	(84.4)	44	(93.6)	
Initial UICC	Missing	1	(0.8)	1	(1.3)		–	0.227
	I	14	(11.3)	7	(9.1)	7	(14.9)	
	II	11	(8.9)	4	(5.2)	7	(14.9)	
	III	12	(9.7)	6	(7.8)	6	(12.8)	
	IVA	52	(41.9)	33	(42.9)	19	(40.4)	
	IVB	15	(12.1)	12	(15.6)	3	(6.4)	
	IVC	19	(15.3)	14	(18.2)	5	(10.6)	
Duration of	median (IQR)	15.1	(7.2–33.1)	10.7	(6.4–30.4)	21.8	(9.8–37.3)	0.186
disease, months								
Extent of	LRR	39	(31.5)	19	(24.7)	20	(42.6)	**0.038**
disease	M1	85	(68.5)	58	(75.3)	27	(57.4)	
Previous	None	10	(8.1)	9	(11.7)	1	(2.1)	0.104
treatments	One	66	(53.2)	44	(57.1)	22	(46.8)	
	Two	40	(32.3)	19	(24.7)	21	(44.7)	
	Three	6	(4.8)	4	(5.2)	2	(4.3)	
	Four	2	(1.6)	1	(1.3)	1	(2.1)	
Type of prior	No prior ChT	56	(45.2)	36	(46.8)	20	(42.6)	0.652
treatment	none	*10*	*(17.9)*	*9*	*(25.0)*	*1*	*(5.0)*	
	PORT	*28*	*(50.0)*	*20*	*(55.6)*	*8*	*(40.0)*	
	RT	*8*	*(14.3)*	*4*	*(11.1)*	*4*	*(20.0)*	
	OP	*10*	*(17.9)*	*3*	*(8.3)*	*7*	*(35.0)*	
	Prior ChT	68	(54.8)	41	(53.2)	27	(57.4)	
	CRT	*14*	*(20.6)*	*9*	*(22.0)*	*5*	*(18.5)*	
	PORCT	*54*	*(79.4)*	*32*	*(78.0)*	*22*	*(81.5)*	
RCT enrollment	No	56	(45.2)	43	(55.8)	13	(27.7)	**0.002**
	Yes	68	(54.8)	34	(44.2)	34	(72.3)	
	1L trial	*68*	*(100.0)*	*34*	*(50.0)*	*34*	*(50.0)*	
	2L trial	*13*	*(19.1)*	*11*	*(32.4)*	*2*	*(5.9)*	
Prior cisplatin	Yes	61	(49.2)	36	(46.8)	25	(53.2)	0.487
	No	63	(50.8)	41	(53.2)	22	(46.8)	
Further	no	97	(78.2)	56	(72.7)	41	(87.2)	0.058
therapies	2L-/3L-ChT	27	(21.8)	21	(27.3)	6	(12.8)	
OS status	alive	3	(2.4)	2	(2.6)	1	(2.1)	0.577
	NCRD	5	(4.0)	2	(2.6)	3	(6.4)	
	CRD	116	(93.5)	73	(94.8)	43	(91.5)	

*P value from Pearson’s Chi-square (χ^2^) tests. PFE, Cisplatin/5-fluoruracil/cetuximab—EXTREME regimen; IQR, Interquartile range; 1L-ChT, first-line chemotherapy; py, pack years; L-/HPSCC, laryngeal/hypopharyngeal squamous cell carcinoma; OSCC, oral squamous cell carcinoma; OPSCC, oropharyngeal squamous cell carcinoma; UICC, tumor stages according to the Union International Contre le Cancer; LRR, locoregional recurrence; M1, distant metastasis; ChT, chemotherapy; PORT, postoperative radiation; RT, primary radiation; OP, surgical therapy; CRT, combined chemo-radio-therapy; PORCT, postoperative chemo-radio-therapy; RCT, randomized controlled trial; 1L trial, first-line controlled trial; 2L trial, second-line controlled trial; 2L-/3L-ChT, second-/third-line chemotherapy; OS, Overall Survival; NCRD, Non-cancer-related death; CRD, cancer-related death. P values from Pearson’s Chi-square tests < 0.05 are in bold.

### Clinical Work-Up for R/M HNSCC

As recommended (2), clinical work-up for R/M HNSCC included clinical examination, ultrasound sonography, contrast-enhanced CT for head and neck and thorax, eventually PET-CT/PET-MRI, followed by a panendoscopy accompanied by taking biopsies before decision-making for treatment in the MDTB. Patient and tumor characteristics, diagnostic procedures, treatment and clinical follow-up were recorded in our Microsoft Access^®^ tumor database (TDB) and OncoFlow^®^ (10, 11).

### CT and PET-CT Imaging

According to clinical guidelines, all patients received a head and neck and a CT scan of the chest during staging. In 2006, a PET-CT became available. An experienced board-certified nuclear-medicine physician and a radiologist analyzed PET-CTs. Sites of tumor involvement were identified visually by enhanced, non-physiologically [^18^F]-FDG uptake.

### Decision-Making Process in the MDTB

The decision-making process in the MDTB followed ASCO and NCCN guidelines (2) and principles published earlier (10–13). Briefly, a radiologist and a nuclear medicine specialist presented all radiological imaging. The MDTB consisting of head and neck and maxillofacial surgeons, a pathologist, an oncologist, a radiation oncologist, and other clinical staff involved in the treatment of head and neck cancer patients discussed the results of diagnostic procedures. Considering the general health and comorbidity of the patient the pre-therapeutic MDTB regarding the guidelines (2) recommended the type of 1L-ChT according to inclusion criteria of open randomized controlled trials (RCTs) or according to fitness for current therapy standards, PFE or other 1L-ChT. For the subgroup of patients receiving 1L-ChT other than PFE, the most relevant RCTs were CeFCiD (NCT02268695), RESGEX (NCT02052960) and ADVANTAGE (NCT00705016) (6–8).

### Immunohistochemistry for P16 and HPV Genotyping

Before decision-making in MDTB and starting therapy, biopsies were taken under general anesthesia and underwent pathological examination. Pathological reports were available for all 124 patients. Besides hematoxylin–eosin (HE) staining, molecular analyses of p16 by immunohistochemistry utilizing the CINtec kit (Roche) were done in oropharynx squamous cell carcinomas (OPSCC) of RCT participants and performed in OPSCC routinely since 2013. Double-stained, p16-positive/Ki67-positive cells or a cutoff level of ≥70% p16-positive OPSCC cells were considered p16+. DNA of p16+ OPSCC was extracted and analyzed for high-risk human papillomavirus utilizing the INNO-LiPA HPV Genotyping Extra kit (Innogenetics) as described earlier (14).

### Statistical Analysis

Overall survival (OS) was the time from initial diagnosis of HNSCC to cancer-related (CRD) or non-cancer-related death (NCRD) censoring patients alive at the end of follow-up (data base lock: 08.02.2021). Survival after 1L-ChT (OS_1L-ChT_) was the time from diagnosis that led to 1L-ChT until death by any cause, censoring patients alive at the end of follow-up or data base lock. We performed a statistical analysis in SPSS 25^®^. We used *Chi-square* tests, paired and heteroscedastic *t*-tests, receiver-operating characteristics (ROC) curves and Fisher’s exact test to investigate the association of clinical characteristics and the outcome of patients receiving PFE or other 1L-ChT. Kaplan–Meier cumulative survival plots and log-rank tests were used to investigate the impact of particular characteristics on OS_1L-ChT_. We analyzed all parameters achieving *P <*0.2 in univariate models in multivariate Cox proportional hazard regression (MCR) models. After checking collinearity, independent predictors for the OS_1L-ChT_ have been identified in MCR applying the step-wise-forward method. For internal validation and to reduce over-optimism based on the effects of random sampling errors, we utilized bootstrapping (1,000 iterations). We considered *P <*0.05 being significant.

## Results

### Patients’ Characteristics

Of 124 R/M HNSCC patients, 77 received PFE (**Table 1**). The frequency of PFE was numerically higher in patients younger than 60 years (68.1% *vs*. 54.5%; *X^2^* = 2.4, *P* = 0.122). Other 1L-ChT regimens applied to 47 patients not receiving PFE were PFE plus docetaxel (TPFE; *n* = 15) according to the CeFCiD trial (6) and other cisplatin-based regimens (*n* = 21 in total, every subgroup *n <*5); 11/47 patients received in 1L-ChT docetaxel plus cetuximab (*n* = 3) or a monotherapy with methotrexate (*n* = 1) or immunotherapy with either cetuximab (*n* = 3) or nivolumab (*n* = 4). ECOG performance status in subgroups receiving PFE or other 1L-ChT did not differ significantly (**Table 1**).

### Patients’ Clinical Course Before and After 1L-ChT

The median time from the initial diagnosis of HNSCC to 1L-ChT was 15.1 months for the total cohort. There was no significant correlation between the time to 1L-ChT and the lifestyle-associated risk factors or patients’ age. Patients receiving surgery followed by postoperative radio-chemotherapy (PORCT; n = 52) had a prolonged median time from curative treatment to 1L-ChT of 30.6 months (95%CI: 21.5–40.2) compared to 10.4 months (95% CI: 0.3–20.4) of patients with other types of curative treatment (radiation, surgery, surgery followed by postoperative radiation; n = 62). Median time from initial diagnosis of HNSCC to death/lost to follow-up (OS) was 25.5 months; median time from start of 1L-ChT to death/lost to follow up (mOS_1L-ChT_) was 8.4 months; 21/124 (16.9%) died within 3 months after starting 1L-ChT (14.3% after PFE, 21.3% after other 1L-ChT regimen). Of 124 patients progressing after 1L-ChT, 27/124 (21.8%) were fit enough to receive either a 2L-ChT or further therapies, 21/77 (27.3%) after PFE, 6/47 (12.8%) after other cisplatin-based regimen. None of the patients treated without cisplatin-based 1L-ChT including all 1L-immunotherapies were fit enough for any 2L-ChT.

### OS_1L-ChT_ After PFE Compared to Other 1L-ChT Regimen

**Figure 2:**
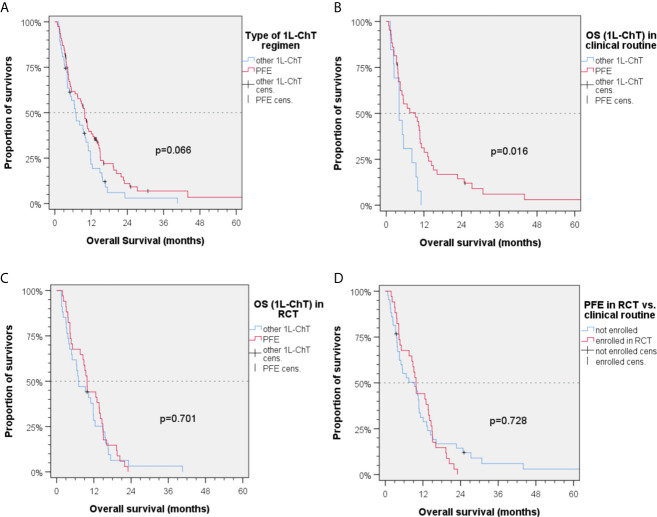
Kaplan–Meier plots for cumulative overall survival (OS_1L-ChT_) measured from diagnosis of incurable recurrent/metastatic head and neck squamous cell carcinoma receiving first-line chemotherapy as indicated; **(A)** OS_1L-ChT_ after PFE according to the EXTREME protocol *vs*. other 1L-ChT regimens; **(B)** OS_1L-ChT_ of patients receiving outside randomized controlled trials (RCT) PFE *vs*. other 1L-ChT regimens; **(C)** OS_1L-ChT_ of patients receiving PFE *vs*. other 1L-ChT within RCT; **(D)** OS_1L-ChT_ of patients receiving PFE in RCT *vs*. outside RCT; *P* values shown are from 2-sided log-rank tests.

In Kaplan–Meier plots utilizing log-rank tests, a difference of 3 months in mOS_1L-ChT_ was identified between patients being treated with PFE and those being treated with other 1L-ChT (mOS_1L-ChT_ (95%CI): 9.8 months (8.1–11.5) *vs.* 6.8 months (3.9–9.7); *P* = 0.066; **Figure 2A**).

### OS_1L-ChT_ after PFE in RCT *Versus* Clinical Routine

In the group of patients not enrolled in first-line RCT (“real world patients”), a significant benefit from PFE was noticed [mOS_1L-ChT_ (95%CI): 9.3 (3.3–15.3) months *vs.* 4.1 (1.8–6.4) months, *P* = 0.016; **Figures 2B** and **4**]. In RCT, other 1L-ChT combined *vs.* PFE showed a similar OS [mOS_1L-ChT_ (95%CI): 7.0 (3.0–11.0) months *vs*. 9.8 (8.7–11.9) months; *P* = 0.701; **Figure 2C**]. OS_1L-ChT_ after PFE outside controlled trials [mOS_1L-ChT_ (95%CI): 9.3 (3.3–11.3) months) was not significantly different from mOS_1L-ChT_ in RCTs (9.8 (8.7–10.9) months; *P* = 0.728; **Figure 2D**]. Of seven long-term survivors within the subgroup of patients treated with PFE in clinical routine (**Figure 2D**), 4/7 were current drinkers, only 1/7 drank >30 g/d alcohol, and 5/7 were current smokers. The median age (56.9 years) was comparable to the median age in the total PFE subgroup (56.6 years, **Table 1**). Five of them (71.4%) had been treated with cisplatin prior to PFE, compared to 46.8% in the total PFE cohort (**Table 1**).

### OS_1L-ChT_ After Other 1L-ChT Regimen

In this subgroup, the enrollment in RCT was predictive for improved OS_1L-ChT_ only in these 34 *vs.* 13 patients (mOS_1L-ChT_ (95%CI): 9.3 (4.7–13.9) *vs*. 4.1 (1.8–6.4); *P* = 0.013). The small number of patients with other 1L-ChT (*n* = 47), however, did not allow to identify further predictors for OS_1L-ChT_ in this subgroup.

### Predictive Factors for OS_1L-ChT_ After PFE

Kaplan–Meier plots showed the number of pretreatments to be important for therapy outcome in general. Patients initially diagnosed in the metastatic or very advanced stage or after two or more pretreatments had significantly shorter OS_1L-ChT_ than those receiving 1L-ChT after one pretreatment (mOS_1L-ChT_ (95%CI): 6.8 (4.2–9.4) *vs*. 9.9 (7.6–12.2) months; *P* = 0.038). Stratified by PFE *vs*. other 1L-ChT, there was still a statistical trend for this finding (**Figure 4**). Patients progressing after cisplatin-based ChT treated with PFE 1L-ChT had prolonged mOS_1L-ChT_ (9.9 *vs*. 6.8 months; *P* = 0.082; **Figure 4**). Cisplatin-based ChT as part of multimodal pretreatment in the curative setting was equally predictive for OS_1L-ChT_ in univariate Cox regression model.

Kaplan–Meier analysis linked outcome and age: the mOS_1L-ChT_ in the age groups (a) ≤49 years (7.6 months, 95%CI: 0.2–15.0), (b) 50–59 years (9.3 months, 95%CI: 7.6–11.0), and (c) ≥60 years (6.8 months, 95%CI: 4.6–9.0) was insignificantly different (*P* = 0.192). Stratified by PFE *vs*. other 1L-ChT, the statistical trend proved to be true and revealed patients aged 50–59 years having the longest OS_1L-ChT_ independent from the type of 1L-ChT applied (mOS_1L-ChT_ (95%CI): 9.8 (7.7–11.9) after PFE *vs*. 8.2 (0.0–17.6) months after other 1L-ChT regimen; *P* = 0.560). There were only 11 *vs*. 8 patients aged ≤ 49 years, the mOS_1L-ChT_ after PFE *vs*. other 1L-ChT was 10.3 (95%CI: 1.6–19.0) months *vs*. 3.3 (95%CI: 0.0–8.0) months (Δ 7.0 months; *P* = 0.754). However, there was a statistical trend in patients ≥ 60 years (30 *vs*. 25 patients) for improved mOS_1L-ChT_ after PFE *vs*. other 1L-ChT of 7.5 (95%CI: 1.6–13.4) months *vs*. 6.4 (95%CI: 3.6–9.2) months (Δ 1.1 months; *P* = 0.082; **Figure 4**). Among PFE-treated patients, we did not see an inferior OS_1L-ChT_ of patients older than 65 years compared to younger patients (21 *vs*. 56 patients; OS_1L-ChT_ (95%CI): 9.9 (1.3–18.5) *vs*. 9.3 (6.9–11.7); *P* = 0.467). Even with a slightly different cut-off point of 60 years (30 *vs*. 47 patients then), we did not see a significant difference neither (OS_1L-ChT_ (95%CI): 7.5 (1.6–13.4) *vs*. 9.9 (8.0–11.8); *P* = 0.974). However, the heterogeneity in response to PFE in older patients is demonstrated by the enlarged 95%CI.

**Figure 3:**
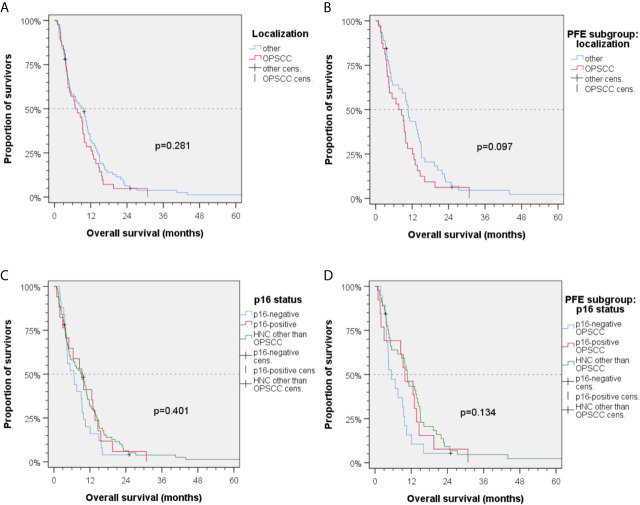
Kaplan–Meier plots for cumulative overall survival (OS_1L-ChT_) measured from diagnosis of incurable recurrent/metastatic head and neck squamous cell carcinoma **(A, C)** in the total cohort and **(B, D)** PFE treated patients. **(A)** OS_1L-ChT_ for OPSCC *vs.* index HNSCC outside the oropharynx; **(B)** OS_1L-ChT_ after PFE for OPSCC *vs*. HNSCC outside the oropharynx; **(C)** OS_1L-ChT_ in p16-negative OPSCC *vs*. p16-positive OPSCC *vs*. HNSCC outside the oropharynx; **(D)** OS_1L-ChT_ after PFE in p16-negative *vs*. p16-positive *vs*. HNSCC outside the oropharynx; *P* values shown are from 2-sided log-rank tests.

Regarding different localizations of the primary site of the R/M HNSCC, a statistical trend for oropharyngeal cancer *vs*. HNSCC outside oropharynx was found in Kaplan–Maier analyses (mOS_1L-ChT_ (95%CI): 6.8 (2.9–10.7) months *vs*. 9.5 (6.6–12.4) months; *P* = 0.281; **Figure 3A**). Analyzing the PFE subgroup (n = 77), this difference was more than 3 months (OS_1L-ChT_ (95%CI): 7.6 (2.5–12.7) *vs*. 10.7 (9.2–12-2); *P* = 0.097, **Figure 3B**). The p16-status was critical for OS_1L-ChT_. As p16-positive (p16+) OPSCC had mOS_1L-ChT_ of 9.3 (95%CI: 4.6–14.0) months comparable with non-oropharyngeal cancer (9.5 (6.6–12.4) months; *P* = 0.784), p16-negative OPSCC had impaired mOS_1L-ChT_ of 6.7 (95%CI: 2.9–10.5) months (**Figure 3C**). Stratified by type of 1L-ChT, we saw impaired OS_1L-ChT_ in p16-negative OPSCC patients even if PFE treated (**Figure 3D**). Considering HPV-driven OPSCC (*n* = 15 p16+ HR-HPV-DNA+ OPSCC out of *n* = 17 p16+ OPSCC) did not result in deviating measures but reduced differences due to enlarged 95% CI and increased *P* values, besides use of sole p16-IHC in clinical routine the reason for reporting results for p16+ OPSCC in **Figures 3** **–** **5**.

**Figure 4:**
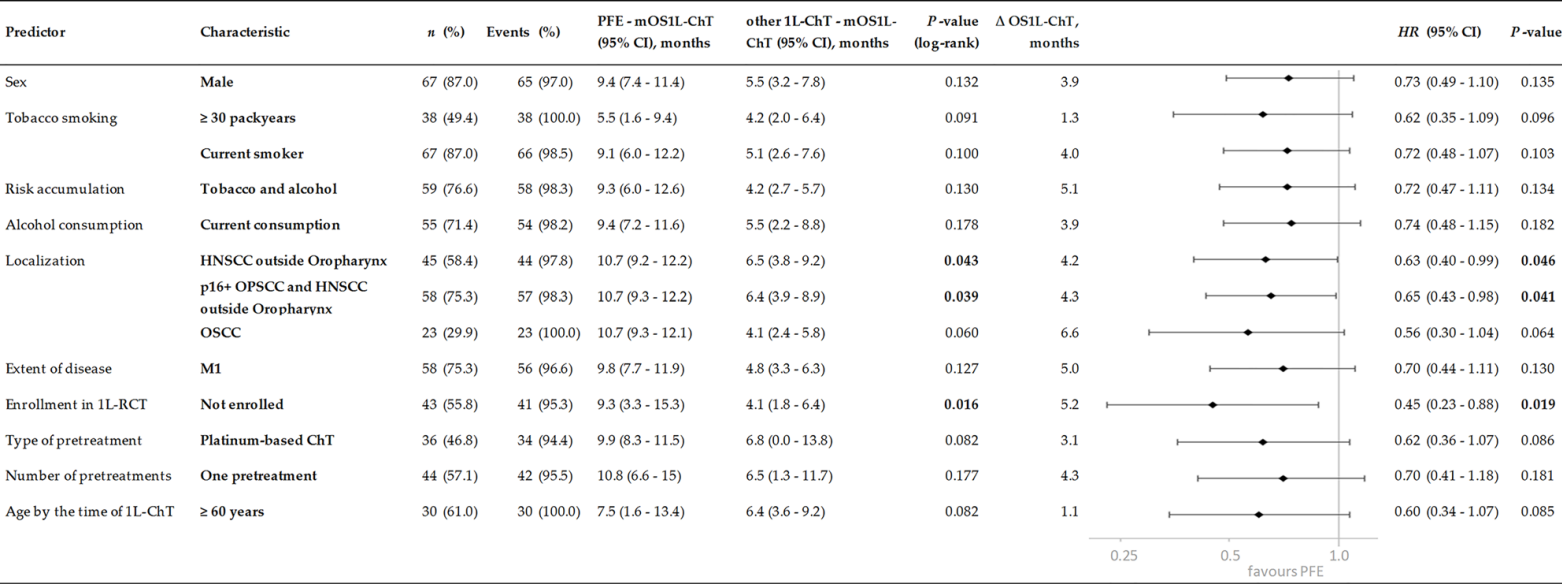
Subgroups of incurable recurrent/metastatic head and neck squamous cell carcinoma benefitting from PFE administered according to the EXTREME protocol by prolonged OS_1L-ChT_, demonstrated by Kaplan–Meier estimates applying log-rank tests and univariate Cox proportional hazard regression. *P* values of significant predictors <0.05 are in bold.

Patients with index HNSCC outside the oropharynx had a significant benefit from PFE *vs*. other 1L-ChT regimens [OS_1L-ChT_ (95%CI): 10.7 (9.2–12.2) *vs*. 6.5 (3.8–9.2) months; *P* = 0.043; Δ 4.2 months; **Figure 4**]. Patients by the time of 1L-ChT diagnosed with distant metastasis (M1) demonstrated an improved benefit from PFE compared to patients with loco-regional recurrence (**Figure 4**). However, we performed sensitivity analyses and excluded all patients that were diagnosed already in a locally very advanced and metastatic stage without any curative option and therefore receiving 1L-ChT as first treatment (*n* = 10). Kaplan–Meier estimates showed a mOS_1L-ChT_ after PFE *vs*. other 1L-ChT of 9.4 (95%CI: 7.8–10.9) months *vs*. 6.5 (95%CI: 4.1–9.2) months for the remaining 114 patients (*P* = 0.163). This compares well to the OS_1L-ChT_ for the total cohort.

The lifestyle-factors tobacco and alcohol showed an impact on outcome (**Figure 4**). There were patients with both risk factors (current or former alcohol consumption and tobacco smoking; *n* = 93) and those without or solely one risk factor (*n* = 26; five patients without information). Both groups demonstrated a benefit from PFE, patients with two risk factors had an impaired mOS_1L-ChT_ but showed a higher benefit from PFE in Kaplan–Meier estimates [mOS_1L-ChT_ (95%CI): 9.3 (6.0–12.6) *vs*. 4.2 (2.7–5.7) months; *P* = 0.130; **Figure 4**]. We found a significant correlation of double-positive risk factor-anamnesis with two baseline characteristics: young patients (≤60 years at 1L-ChT; *Pearson’s r* = 0.272; *P* = 0.003) and male patients (*Pearson’s r* = 0.288, *P* = 0.002) did more often belong to the group with both risk factors.

### Multivariate Cox Proportional Hazard Regression for Outcome

The MCR model for OS in the total cohort achieving highest significance (*X^2^* = 21.7, *P* = 0.001) included five independent risk factors: the number of pretreatments and pack years smoking history, alcohol consumption status, index HNSCC of the oropharynx, and type of 1L-ChT (**Figure 6**). Bootstrapping revealed these factors to be predictive for OS_1L-ChT_. The stepwise forward method for building the MCR failed to detect any predictive value of the patient’s age by the time of 1L-ChT, TNM at first diagnosis or even enrollment in a first-line RCT for OS_1L-ChT_. Interestingly, having a p16+ OPSCC was also not predictive for improved outcome, and MCR including either p16 positivity or p16 negativity as covariate had reduced significance compared to MCR including OPSCC as covariate; therefore OPSCC summarizing p16+ and p16- OPSCC remained in the MCR.

In MCR model for the PFE subgroup (n = 77; *X* ^2^ = 15.0, *P* = 0.002), three risk factors were found to be predictive for OS_1L-ChT_ after PFE. Cisplatin-based CRT/PORCT prior to PFE (*HR* (95%CI): 0.67 (0.42–1.09); *P* = 0.106) was beneficial, index OPSCC (*HR* (95%CI): 1.61 (0.97–2.68); *P* =0.066) and alcohol consumption ≥ 30 g/d (*HR* (95%CI): 2.04 (1.22–3.41); *P* = 0.007) predicted impaired OS_1L-ChT_.

### Identification of PFE Long-Term Survivors

**Figure 5:**
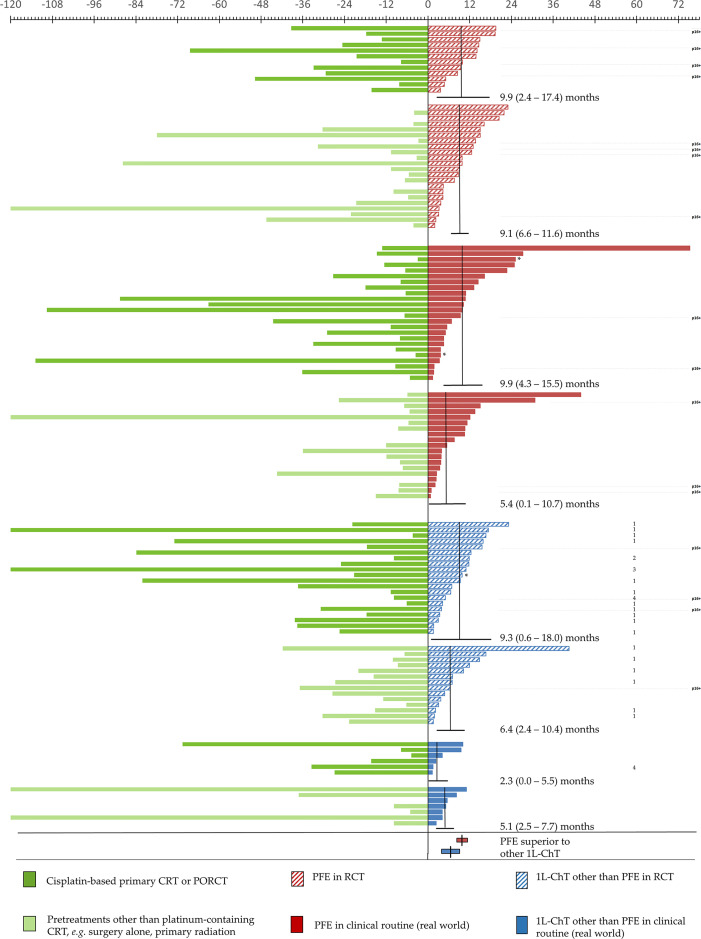
Individual outcome of 124 patients with incurable recurrent/metastatic head and neck squamous cell carcinoma (R/M HNSCC) receiving various first-line chemotherapy regimens are depicted according to overall survival measured from diagnosis of R/M HNSCC til death (OS_1L-ChT_). Patients are shown sorted stratified according to 1L-ChT, either EXTREME-regimen (PFE, red; *n* = 77) or other 1L-ChT (blue; *n* = 47) and treatment either within randomized controlled trial (RCT; shaded) or in clinical routine (“real world setting”, full). Type of prior treatment in curative attempt is indicated in dark green (cisplatin-based chemo-radiation (CRT) or post-operative radio-chemotherapy (PORCT)) *vs*. light green (other or no pretreatment); time from initial diagnosis of HNSCC until diagnosis of incurable disease requiring 1L-ChT is shown in the left panel, OS_1-ChT_ in the right panel according to the upper scale showing time in months. The horizontal lines indicate mOS_1-ChT_ (95% confidence interval). Median and 95%CI of OS_1l-ChT_ of PFE *vs*. other 1L-ChT in the total cohorts are shown in the lower rows. *censored: alive at last follow-up (*n* = 3); 1, CeFCiD (6); 2, ADVANTAGE (7); 3 RESGEX (8); 4, TPExtreme (9); p16+, p16+ OPSCC.

**Figure 5** shows individual OS_1L-ChT_ in R/M HNSCC stratified according to PFE *vs.* other 1L-ChT either in treatment within RCT or in clinical routine providing “real world evidence”. According to identification of prior cisplatin-based CRT or PORCT as significant OS_1L-ChT_ predictor, we further stratified these groups by cisplatin-based CRT or PORCT *vs.* other pretreatments. The improved outcome of certain PFE-treated R/M HNSCC patients allowed further investigations in the subgroup surviving more than 11.3 months, the upper bound of 95%CI for mOS_1L-ChT_ in PFE-treated patients. These 28 individuals had a median age (57.3 years) comparable to the total cohort of PFE patients (*n* = 77, 56.6 years). They were quite similar to the total PFE cohort respective to sex (17.9% female), type of prior treatment (50% cisplatin-based CRT/PORCT) besides slightly lower median exposure to risk factors (22 pack years in 64.3% current smokers, as well as 64.3% current alcohol consumers; **Table 1**). Even the 12 RWE-PFE patients with OS_1L-ChT_ above 95%CI OS_1L-ChT_ in the CRT/PORCT and “other” subgroups (10.7 and 15.5 months, respectively) had a similar median age at the time of initial diagnosis of HNSCC (57.4 years) compared to median age in the PFE subgroup (56.6 years, **Table 1**). Eleven of these 12 patients received PFE at first recurrence, one (8.3%) was treated with PFE at second recurrence. As one curative treatment prior to any 1L-ChT is an independent predictor for improved OS_1L-ChT_ in the total cohort, this might be causative involved in their prolonged OS_1L-ChT_. However, only 2/12 (16.7%) of RWE-PFE long-term survivors had a current alcohol consumption >30 g/d, pointing to the absent detrimental impact of maintained alcohol consumption on OS_1L-ChT_ in most of RWE-PFE long-term survivors. Interestingly, smoking history and adhering to tobacco smoking may also play a role as only seven of these 12 long-term survivors (58.3%) were current smokers, and the median cumulative nicotine exposure was 25 pack years and somewhat lower compared to the total PFE cohort (**Table 1**). The proportion of p16+ OPSCC was higher in RCT; their OS_1L-ChT_, however, was not superior compared with R/M HNSCC localized outside the oropharynx (**Figure 3C**).

**Figure 6:**
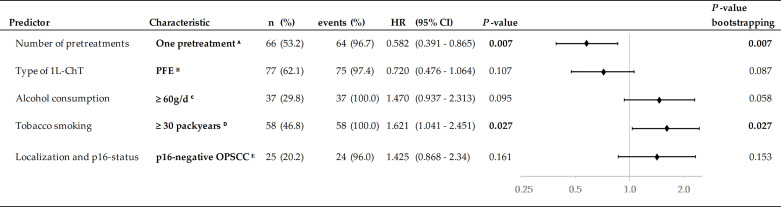
Predictors in multivariate Cox proportional hazard regression (HR) and 2-sided *P*-values from internal validation using bootstrapping applying 1,000 iterations. Significant independent predictors *P* <0.005 are in bold. ^A^ Reference: 1L-ChT at initial diagnose or ≥2 pretreatments; ^B^ Reference: other 1L-ChT regimen; ^C^ Reference: <60 g/d; ^D^ Reference: <30 pack years; *E* Reference: HNSCC outside oropharynx.

### Identification of Long-Term Survivors in Other 1L-ChT Regimens

As enrollment in RCT was predictive for improved OS_1L-ChT_ only in 34 *vs.* 13 patients (see *OS_1L-ChT_ after other 1L-ChT regimen*) we were interested in long-term survivors in this subgroup. According to numbers in the right panel of **Figure 5**, PFE-based regimens containing an additional (investigational) drug, for instance docetaxel (TPFE) in the CeFCiD trial [labeled 1 (6)], cilengitide in the ADVANTAGE trial [labeled 2 (7)], or replaced cetuximab by glycosylation-modified cetuximab in the RESGEX trial [labeled 3 (8)], long-term survivors were only seen after PFE-based 1L-ChT. However, the outcome observed in such intensified PFE-based 1L-ChT did not improve outcome in general at least in our cohort as it is obvious that a huge heterogeneity exists in this regard.

## Discussion

According to several lines of evidence, our monocentric study comprises a sufficient number of R/M HNSCC receiving 1L-ChT to show outcome differences dependent on a number of well-defined covariates. The mOS_1L-ChT_ in our sample is comparable to the survival times found in prior trials (6, 7, 15). Therefore, the subgroups with and without benefit from PFE identified in our study confirm the existence of certain subgroups already described (3). Uni- and multivariate analyses demonstrated that the number of pretreatments, consumption of alcohol and/or tobacco smoking as well as localization of the index cancer and patients’ age have a certain effect on OS_1L-ChT_. When treated with PFE in particular, predictive covariates are mostly the same. However, our study provides evidence that prior intensified treatments making use of cisplatin-based CRT and especially cisplatin-based PORCT do not negatively affect survival in PFE but rather improve OS_1L-ChT_. Indeed, prior cisplatin-based CRT or PORCT appeared to be an additional independent predictor for significant prolonged OS_1L-ChT_. These findings from multivariate Cox regression analyses may contribute to the ongoing discussion about a potential negative impact of treatment escalation in the curative setting on further therapies and the possibility to re-challenge R/M HNSCC with cisplatin when progressing after cisplatin-based curative treatment. As the median time from curative treatment with surgery followed by cisplatin-based PORCT to 1L-ChT (*n* = 52) was 30.6 months (95%CI: 21.5–40.2) and substantially longer (*P* = 0.005) compared to 10.6 months (95%CI: 5.0–16.3) of patients without prior treatment or other types of prior curative treatment, and these cisplatin-pretreated R/M HNSCC patients had the highest benefit from PFE, treatment escalation in presence of risk factors in the curative setting improves outcome and does not reduce OS_1L-ChT_ if PFE is used. As, additionally, a 2L-ChT could be applied in a higher frequency after PFE as compared to other 1L-ChT, treatment escalation in the curative setting *via* cisplatin-based PORCT whenever high risk for relapse/recurrent disease (more than two disease-positive neck nodes, extracapsular extension of neck nodes, positive or narrow resection margins below 5 mm) is detected appears to be warranted.

Our results confirm OS data for PFE including subgroup analyses obtained in the landmark phase-III RCT EXTREME (3). Comparing outcome of PFE with PF, Vermorken et al. (3) showed in univariate models that patients ≥ 65 years demonstrate a minor benefit from PFE compared to younger patients. Our retrospective study comprises only 21 *vs*. 15 R/M HNSCC patients ≥65 years receiving PFE *vs*. other 1L-ChT regimen. We have not seen an inferiority of PFE in this subgroup compared to patients <65 years. By performing this analysis with a slightly different cut-off point of 60 years (30 *vs*. 15 patients), we found no evidence for an inferiority of PFE neither.

The recently published ELAN-FIT trial by Guigay et al. (16) showed a mOS_1L-ChT_ of 14.7 months (95%CI: 11.0–18.2) after PFE for patients aged 70 and older and ECOG performance status 0 or 1. The impact of age and its influence on PFE efficacy and risk will be probably important in future trials. However, we found no evidence in our cohort for calendar age alone being the most relevant eligibility criterion for PFE, provided good general health (ECOG 0 or 1). PFE is only approved for ECOG 0 and 1 patient presenting, so the MDTB made the decision for either offering participation in a 1L-ChT RCT or 1L-ChT treatment in the routine setting only provided good general health as reflected by ECOG 0 or 1. Consequently, our sample mainly included “fit” patients in our retrospective trial to ensure comparability. As Guigay et al. (17) showed, “unfit” patients may be eligible for PFE or comparable regimens after a comprehensive geriatric assessment. By performing RCTs after a geriatric assessment, there could be more evidence about the impact of calendar age *vs*. biological age on treatment eligibility and potential benefit in older patients.

Referring to Guigay et al. (9), the TPExtreme (TPE; docetaxel, cis- or carboplatin, cetuximab) 1L-ChT regimen is beneficial when followed by ICB in 2L-ChT. As retrospectively found, TPE outperformed PFE only in this treatment sequence. Due to our small sample of 124 patients collected over years and only two TPE patients unfit to receive 2L-ChT after recurrence, there are no such patients in our cohort.

Patients in the PFE subgroup had a longer mOS_1L-ChT_ – independently on the following 2L-ChT— than patients treated with other 1L-ChT in our analysis. As all studies demonstrated the lasting value of PFE, we recommend—against the often suggested alternative use of TPE as unproblematic replacement for PFE to avoid potential dihydropyrimidine dehydrogenase- (DPD-) toxicity—rather DPD testing according to established guidelines (18) so that R/M HNSCC patients still can benefit from PFE. As only one RCT demonstrated an improved OS of R/M HNSCC in the minor subgroup of patients treated sequentially first with TPE followed by ICB over PFE followed by ICB in a retrospective analysis (17), it might be too soon to change 1L-ChT of R/M HNSCC in absence of a positive phase III RCT demonstrating superiority of TPE over PFE. Moreover, we were unable to see a benefit from TPE as only 2/124 patients received TPE, and both (indicated with four in **Figure 5**) had a rather impaired outcome below the mOS_1L-ChT_. Without replication of the findings by Guigay et al. (9) in such a phase-III RCT the TPExtreme-ICB treatment sequence so far remains experimental at best.

Today, no published data for the efficacy of PFE for R/M HNSCC progressing under 1L-ICB are available. The question if patients failing on curative treatment involving ICB thereafter progressing and requiring 1L-ChT should preferentially be treated with PFE is not yet completely clear. However, we expect that PFE can benefit a substantial proportion of such R/M HNSCC.

Regarding the influence of HPV-status on OS_1L-ChT_, we have seen an impaired OS_1L-ChT_ in patients suffering from a p16-negative OPSCC compared to patients with a p16+ OPSCC or index HNSCC outside the oropharynx. This is in line with former findings (19, 20). Based on the study by Mehra et al. (19) showing an improved OS in p16+/HPV+ R/M HNSCC patients, Vermorken et al. (20) performed a retrospective analysis of data from the EXTREME trial (3) and found a p16+/HPV-prevalence and p16+/HPV-related OS_1L-ChT_ similar to our findings. There is an ongoing discussion about the influence of HPV on survival in R/M HNSCC. In contrast to Mehra et al. and Vermorken et al., Szturz et al. (21) found in a meta-analysis of four prospective RCT that HPV-related (p16+ or HPV-DNA+) tumors barely responded to EGFR-directed monotherapy, whereas improved response rates were only observed in HPV-negative cases. Since we did not observe detrimental effects by p16 positivity on OS_1L-ChT_ no matter if EXTREME or other regimens were applied, but OS_1L-ChT_ was strongly reduced in oropharyngeal R/M HNSCC and even further reduced in p16-negative cases, our study highlights the importance of further investigations in this field. The poorest OS_1L-ChT_ in oropharyngeal R/M HNNSCC could be linked to the proximity to essential cervical structures including arteries and their infiltration. Therefore, R/M HNSCC with rather reduced infiltrating growth patterns and without vascular infiltration may have prolonged OS_1L-ChT_ independent from being HPV-related. Additionally, distance of the R/M HNSCC from vital vessels might prolong the time to life-threatening destruction of indispensable organs and critical bleeding events including arterial blowout leading to death.

During the time period analyzed in this retrospective study, therapy guidelines for R/M HNSCC have changed. Nowadays, and according to KEYNOTE-048 trial (4), immune checkpoint blockade (ICB) by pembrolizumab is declared new standard of care for patients with CPS >20 or ICB-PF combination for patients with CPS >1 to ≤20. According to KEYNOTE-048 investigators, PFE remains standard of care for CPS ≤1. Consequently, PFE may be 1L-ChT standard for this subgroup and 2L-ChT option for patients progressing after ICB. However, as we confirm data from the EXTREME trial (3), especially male patients, subgroups accumulating more lifestyle-associated risk factors, and those with their index HNSCC outside oropharynx still benefit the most from PFE. KEYNOTE-048 subgroup analyses (4) addressed this issue showing that patients <65 years and ≥65 years do not differ in benefit from pembrolizumab ± chemotherapy. There was no significant difference between never and former/current smokers. It may be interesting to conduct further analyses to see if there are any differences in OS depending on patients’ characteristics described here (**Figure 5**).

Unlike ICB in the KEYNOTE-048 trial, ICB with durvalumab (PD-L1 inhibitor) ± tremelimumab (CTLA-4-inhibitor) in the KESTREL phase III trial failed to meet the primary endpoint of improved OS compared to PFE. As AstraZeneca reported this result just recently [2021-02-05 (22)] and a peer-reviewed paper on KESTREL is still not published, it might be too soon to rank any ICB in general over PFE. At least, PFE should be considered standard for all 1L-ChT not belonging to the CPS >1 subgroup of R/M HNSCC patients.

Argiris et al. (23) showed an improved response rate and progression-free survival by adding the anti-VEGF antibody bevacizumab to chemotherapy. This may provide evidence for a benefit by targeted therapies other than EGFR- or PD-L1-inhibitors combined with PF. However, acute toxicity appeared to be increased if PF and bevacizumab were used in 1L-ChT, and the gain in OS compared to PF rather limited (18).

Discussing their KEYNOTE-048 results and referring to retrospective trials (24, 25), post-pembrolizumab sensitization of R/M HNSCC to a subsequent therapy with PFE was mentioned by Burtness et al. (4). This highlights the potential importance of 2L-PFE applied after 1L-ICB in the future. In the light of ICB applied within multimodal treatment regimen in the curative setting, *e.g.* during induction-chemotherapy for larynx-organ preservation or ICB as component of adjuvant therapies after curative resection and in postoperative maintenance, we are convinced that PFE will have a dominant role as 1L-ChT also in the future (26, 27). In context of earlier investigations highlighting improved outcome after increased utilization of PORCT in treatment of L/HSCC (23), prolonged OS_1L-ChT_ through PFE after cisplatin-based PORCT may at least partially have contributed to the welcome impact of indication shift towards increased use of cisplatin-based PORCT according to Bernier and Cooper (12, 13) on heightened OS time (28).

In our study, 14.3% of the patients died within 3 months after starting PFE. These figures compare well to 17.1% found by Vermorken et al. (3). The majority of early deaths observed in our cohort occurred outside RCTs (72.7% *vs*. 27.3% of all fatalities during PFE treatment). Treatment in clinical routine apart from adherence to the complete checklist of eligibility criteria as required to enter any of the RCT as well as survivorship bias may have potentially contributed to this situation. However, outcome in RCT *vs.* “real world” was not significantly different overall. Reproducibility of survival benefit of certain subgroups independent from RCT participation shows that RCT results are representative for the outcome achieved by PFE even in clinical routine. Subgroup analyses of the seven long-term survivors within the subgroup of RWE-PFE treated patients allude to the impact of risk factors on survival. Those seven patients barely drank alcohol but received PFE after cisplatin-based CRT/PORCT. The overall well-comparable or even slightly improved outcome in RWE compared to RCT PFE-treated R/M HNSCC patients demonstrates an unprecedented translation of findings from RCT into routine results with high concordance.

Only a minority of R/M HNSCC patients treated in other 1L-ChT RCT demonstrated superior OS_1l-ChT_ from a further intensified PFE-based regimen, whereas most had inferior OS_1l-ChT_ compared to PFE (**Figure 5**). However, the only long-term survivors detected among other 1L-ChT received an intensified PFE-based regimen. Unfortunately, the frequency of patients without benefit from treatment escalation was found to be higher than those with prolonged OS_1L-ChT_. Increased toxicity as reported also in (6) and (7) may have essentially contributed to this finding by causing detrimental effects. Further investigations to distinguish long-term survivors and those unsuitable for treatment escalation beyond the use of PFE appear to be warranted.

There are limitations of our study. Our retrospective monocentric study involved 124 R/M HNSCC patients including 77 treated with PFE. However, this case number was sufficiently large enough to elucidate some independent predictors for outcome and to confirm the existence of the earlier described subgroups of R/M HNSCC patients. Moreover, we did not find any significant survival differences between patients receiving PFE in- or outside the numerous first-line RCT arguing for a representative mixture of patients that at least in our clinic remained stable over two decades, a consistency in decision-making for usage of PFE in 1L-ChT for R/M HNSCC, and improved outcome achieved through PFE. Therefore, the effects detected in our sample demonstrate stability over time and confirm the initial findings from the EXTREME trial (3) being representative for good outcome after PFE in general. A strength of our study is the complete follow-up and the multivariate analyses including bootstrapping for internal validation of independent predictors to avoid over-optimism in interpretation of our findings.

## Conclusions

This retrospective study highlights the lasting value of the triplet cisplatin, 5-fluoruracil, cetuximab (PFE) not only as comparator treatment within randomized controlled trials (RCT) but also—and independent on the age of R/M HNSCC patients—in clinical routine. Interestingly, we found no evidence for a negative impact of prior intensified treatments making use of primary or postoperative cisplatin-based chemo-radiotherapy on overall survival following first-line chemotherapy but rather improved outcome in this subgroup achieved by PFE independent from participation in RCT or applied in the “real world” setting. Demonstrating again the high value of PFE in first-line chemotherapy, this effective treatment should not be replaced by treatments that failed to demonstrate superiority in RCT. PFE should hence remain standard for first-line chemotherapy at least in patients not belonging to the well-defined subgroups of recurrent/metastatic head and neck squamous cell carcinoma eligible for pembrolizumab or PF plus pembrolizumab according to KEYNOTE-048 (4).

## Data Availability Statement

The raw data supporting the conclusions of this article will be made available by the authors, without undue reservation.

## Ethics Statement

The studies involving human participants were reviewed and approved by The Institutional Human Ethics Committee of the University Leipzig (votes 201-10-12072010 and 202-10-12072010). The patients/participants provided their written informed consent to participate in this study.

## Author Contributions

Conceptualization, GW. Data curation, KL, MP, TW, and GW. Formal analysis, KL and GW. Investigation, KL and GW. Methodology, GW. Project administration, GW. Resources, AD and GW. Validation, GW. Visualization, KL and GW. Writing – original draft, KL and GW. Supervision, SW, AD, VZ, and GW. Writing – review and editing, KL, MP, TW, SW, AD, VZ, and GW. All authors contributed to the article and approved the submitted version.

## Conflict of Interest

The authors declare that the research was conducted in the absence of any commercial or financial relationships that could be construed as a potential conflict of interest.
